# What do patients really want? An in-depth examination of patient experience in four Australian hospitals

**DOI:** 10.1186/s12913-019-3881-z

**Published:** 2019-01-15

**Authors:** F. Rapport, P. Hibbert, M. Baysari, J. C. Long, R. Seah, W. Y. Zheng, C. Jones, K. Preece, J. Braithwaite

**Affiliations:** 1 Australian Institute of Health Innovation, Macquarie University, 75 Talavera Road, North Ryde, Sydney, NSW 2113 Australia; 2 St. Vincent’s Health Australia, 340 Albert Street, East Melbourne, VIC 3002 Australia

**Keywords:** Patient experience, Patient satisfaction, Focus groups, Communication and hospital environment, Discharge

## Abstract

**Background:**

Patient satisfaction is an important outcome measure guiding quality improvement in the healthcare setting while the patient-centred care movement places increasing importance on patient engagement in clinical decision-making. However, the concept of patient satisfaction is not clearly defined, and beliefs of patients are not always evident in health surveys. Researchers rarely follow up on surveys to explore patient views and what they mean in greater depth. This study set out to examine perceptions of hospital care, through in-depth, qualitative data capture and as a result, to gather rich, patient-driven information on user experience and satisfaction in the Australian healthcare setting; and identify influencing factors.

**Methods:**

Focus groups were undertaken in four St Vincent’s Health Australia (SVHA) hospitals in 2017 where participants discussed responses to eight questions from the Press Ganey Patient Experience Survey. Thirty people who were inpatients at SVHA.

**Results:**

Good communication and high-quality information at arrival and discharge were found to be important to patients. Communication breakdown was also evident, further exacerbated by a range of environmental factors such as sharing a room with others. Overall, patients’ felt that while their spiritual needs were well-supported by the hospital staff at all SVHA hospitals, it was the clinical teams prioritised their emotional needs. Good communication and environments can improve patient experience and follow-up at home is vital.

**Conclusions:**

Patient-centred care needs careful planning with patients involved at entry and exit from hospital. Focused communication, environmental changes, attending to complaints, and clearer discharge strategies are recommended.

## Background

The patient-centred care movement has placed increasing importance on patient engagement in clinical decision-making, monitoring healthcare, and legitimising health policy via consumer input [1, 2]. Patient experience of healthcare provision, including patient satisfaction, has become an important outcome measure for healthcare organisations, and a mechanism to guide quality improvement [3, 4]. However, patient experience, including patients’ satisfaction or dissatisfaction in services, is not a clearly defined concept [1, 3, 5, 6], and inferences that are often made from survey results may not accurately reflect the true beliefs of service users or be an accurate measure of the services themselves [7].

Research has found little variation in responses to standard questionnaires about care quality, with typically 85–90% of patients found to be ‘satisfied’ [8]. While some patients might critically evaluate their care, others are willing to allow care to be of quite poor quality before expressing dissatisfaction [9]. Thus, a ‘satisfied’ response can indicate different levels of satisfaction for different people. Gaining an in-depth understanding of how satisfaction is defined and described by patients is likely to lead to a deeper knowledge of patients’ experiences and act as a springboard for more meaningful changes to be made to improve services.

### Aims of the research

The study’s aims were to: 1) examine patient perceptions of hospital care and satisfaction with services, in order to understand the user experience in four Australian hospitals, and 2) identify factors that influence patients’ and carers’ reporting of positive or negative experiences in hospital.

## Methods

### Setting

#### St Vincent’s health Australia

Data were collected between May and October 2017 at the facilities of one of the largest healthcare provider organisations in Australia: St Vincent’s Health Australia (SVHA). SVHA has a strong commitment to a patient-centred approach to care, underpinned by the principle that all patients should feel welcomed, valued and safe’ and that patient-centred care enhances patient experience with care services, drives excellence in practice, and benefits all patients, irrespective of background or socio-economic status [10].

SVHA has been providing healthcare services in Australia for 160 years, since it was founded by the Catholic Sisters of Charity in 1857. It has 36 facilities spread across public and private hospitals, aged-care facilities, and co-located research institutes and partner facilities across three states, Victoria, New South Wales, and Queensland [10].

#### The press Ganey patient experience survey

In order to fulfil the aims of the study and examine patient experience and satisfaction with hospital care we utilised the Press Ganey Patient Experience.

Survey (Press Ganey Associates, Inc., South Bend, IN) to frame our focus group questions. The survey, which SVHA administers to all patients after discharge, is structured with background demographic questions and 84 Likert-style items on which patients report their experience of care and of the facility.

To extend knowledge beyond survey responses, the study focussed on eight items and seven corresponding prioritised themes from the survey (Table 1). The items were chosen because SVHA consistently identified these as improvement priorities.

**Table 1:** Questionnaire items which were the focus of the study

Press Ganey number and questionnaire item	St Vincent’s corresponding prioritised theme
I3. Degree to which hospital staff addressed your emotional and spiritual needs	1.Address emotional and spiritual needs
H1. Extent to which you felt prepared/ready to be discharged	2.Prepared you for discharge
I10. How was the communication/coordination between staff looking after you? E3. Communication between the doctor and nurses regarding your care	3.Communication between all staff
I6. Staff efforts to involve you in decision making about your care and treatment	4.Include patient in decisions about treatment
G4. Extent to which staff involved your family or caregiver in decision making	5.Involve family in decision making
G5. Extent to which staff communicated with your family or caregiver	6.Staff communicated with your family
I4. Response to any patient concerns and/or complaints made during your stay	7.Response to concerns/complaints

## Study design

### Focus groups

Focus groups were conducted with patients (and their family members or carers if they wished) who had spent time as inpatients at one of four SVHA hospitals (Table 2). Participants were asked questions and discussed them together under the guidance of two experienced focus group facilitators. Four focus groups were conducted: Public Hospital Site A, from one city; Public Hospital Site B and Private Hospital Site B from a second city; and Private Hospital Site C, from a third.

**Table 2:** Focus group participants by hospital

	Total participants	Number of males and females
Public Hospital Study Site A	10	7 males, 3 females
Public Hospital Study Site B	7	5 males, 2 females
Private Hospital Study Site B	4	3 males, 1 female
Private Hospital Study Site C	9	5 males, 4 females

### Recruitment

SVHA obtained the names and phone numbers of respondents who indicated at the time of completing the Press Ganey Survey that they agreed to be contacted for a follow-up. To be included in the study, patients had to have been an inpatient at one of the hospital study sites in the 6 months prior to data collection and did not have a current formal complaint with the hospital. We enabled a maximum six-month window for recruitment so that we could be as comprehensive as possible with our recruitment strategy. This also allowed us to undertake the extensive preparatory work involved in contacting patients and arranging focus groups to suit participants across three different states. Researchers randomised the patient list, and contacted eligible patients in each hospital, inviting them to take part in a focus group in their local area. Recruitment targeted 15 patients per group. This accounted for expected attrition rates of 20 to 50%; while 8–10 patients is the optimal number of attendees for focus group participation [11]. Those who agreed to participate were sent an information sheet and consent form and were re-contacted one week prior to the focus group to confirm their attendance. Participants were offered AUD$20 (USD$14.40, GBP£11.10) as a contribution to transport and parking costs.

### Data collection procedures

Focus groups were held in a private meeting room at each hospital site. All participants provided written informed consent and agreed to be audio-recorded. One focus group facilitator worked according to an interview schedule to gain group responses to all questions. The other assessed how people interacted and noted group dynamics, processes and coherence [12]. Each focus group discussed four of the eight items – that is, each item was independently discussed by two focus groups [13]. Each focus group ran for an average of one and a half hours. We kept questions broad and chose not to single out any one aspect of hospital organisation or procedure for undue scrutiny. We did this so that we could concentrate on patients’ views, and so as not to pre-empt which aspects of care, such as complex handover arrangements, may be the influencing factors affecting satisfaction with services.

#### Data analysis

Audio-recordings were transcribed verbatim and along with observer notes, were de-identified for review by five study team members. The team worked consistently together to ensure a coherent response to data analysis, without undue, single-researcher influence [14]. Each member read individual transcripts to identify the main issues arising from the items in Table 1. A thematic analytic framework provided the rubric for the analysis, which was adapted from an approach applied by Guest and colleagues [14]. Main issues were noted and coded [10], while the framework included people’s responses presented in positive, negative or neutral terms. Verbatim quotations and patients’ comments on the wording of the survey items assisted with sense-making. Each of the eight items was given equal weight and consideration. The framework was developed iteratively and enriched after each group session, until a comprehensive picture was attained. The group ensured consensus opinion was reached and derived clear descriptive presentations of the full dataset.

## Results

Twenty males and 10 females of mixed ages participated in a focus group and they were spread across the four study sites (Table 2).

When data were analysed, a number of the concepts and experiences explored were shared between survey items. For example, communication with staff was a key concept in not only discharge planning, but also liaising with family members. Some items elicited very similar comments and experiences, for example: ‘*extent to which staff involved family in decision-making*’, and: *‘extent to which staff communicated with your family’.* Consequently, results are presented according to the most relevant themes and linked items, supported by verbatim quotations to provide an in-depth understanding of people’s views of all four hospitals. Eight themes are discussed, the seven themes from Table 1 and an additional theme: “Skills and behaviours” that emerged.

It should be noted that there was overlap in the way that patients recalled salient events, be they positive or negative across themes. Their recollections of events were overriding factors that influenced their views on the rest of their inpatient stay. This was borne out not only in their response to survey questions, but also in their response to focus group questions regarding their experiences of care, and whether or not care was considered to be patient-centred. In addition, a patient’s perception of the severity of their illness, how long they stayed in hospital, and how vulnerable they felt during their stay, affected their perceptions of their dependency on staff during their stay. This clearly influenced their answers to questions about communication and support from staff, particularly in reporting negative experiences.

### Results by theme

#### Theme 1: Addressing emotional and spiritual needs

There were 16 participants who contributed to discussions about whether they felt their emotional and spiritual needs were met. They understood there to be a clear division between the two types of needs. Patients appreciated that this survey item was about how much empathy was shown toward them and how engaged staff were, as well as whether patients’ emotional and spiritual needs were met.

Only three of the 16 participants said they had any spiritual needs, while others noted that they were not *‘*religious’. Nevertheless, participants felt it was important that spiritual support had been readily available even if they did not need it, and that staff handled spiritual support sensitively:

*It's* [spiritual support] *certainly not pushed down your throat or anything like that, you know.* [P9 Public Hospital Site C]

Participants concentrated instead on emotional needs, whilst acknowledging that emotional distress was to be expected as part of a hospital admission:

*Basically, when you come into hospital, anybody who says they’re not distressed or they’re not in a – not so much a panic mode, but their mood is not upbeat … so sometimes it will get to a point where you require some emotional support*. [P2 Public Hospital Site B]

Recalling positive and negative experiences, and therefore in relation to aim 2, emotional fulfilment was described as feeling that staff were empathetic and engaged, nurses and doctors were attentive, and all staff (from cleaners through to agency and senior staff) were caring and courteous. The positivity of many of the staff members was widely acknowledged, with staff from junior to senior staff, commended for their attention:

*Well I found the cleaners even asked you if they could clean the room.* [P7 Private Hospital Site C]

Patients wanted to know what was going to happen next, and to be reassured that they would be looked after as a person: ‘*not as a number*’ [P4 Public Hospital Site B]. However, when emotional needs were not met, negative consequences resulted:

*She [the nurse] was huffing and puffing and saying: ‘Oh alright’. So that was – basically, I was in hospital for a month and there was one person on the whole ward and it was the day where I was quite miserable, that didn’t make my life feel very, very happy.* [P4 Public Hospital Site B]

Sharing a room with other patients was viewed as emotionally stressful for some participants, while some felt staff disengaged when distress was expressed. A participant gave an example of a doctor who they perceived lacked empathy:

*One particular doctor… he was very difficult and very offensive, but* [P7] *was able to convince him that I needed some sort of help … so he gave in and they kept me in hospital.* [P6 Public Hospital Site A]

Other negative issues reported by participants concerned lack of information for patients and feeling powerless in sharing the decision-making. This was exacerbated by seeing nurses and doctors talking amongst themselves and feeling excluded:

*I knew that I would be on a clinical pathway, which I certainly was … but that didn’t always match up with the person-centred care that I had been led to believe was practiced in hospital. Whether clinical pathways actually suited me as a person didn’t get addressed ... If I had been asked I would have said, no way, but I wasn't asked … you're very vulnerable.* [P4 Private Hospital Site C]

Participants also talked about factors that influenced whether or not their emotional needs were met, and it was clear that they saw ‘emotional resilience’, and hence the need for emotional support, to be dependent on how ill they were, and consequently, how vulnerable. In addition, how long they stayed in hospital, the degree to which they wanted to be involved in their own care, and the people with whom they had to share a room appeared to be integral to resilience.

#### Theme 2: Preparing for discharge

The item relating to the extent to which patients felt ready for discharge was discussed by 14 participants. They discussed needing to feel physically well enough to be prepared to go home:

*You should be going home just feeling comfortable and you’ve had, not a good experience, but you’ve had an experience that was successful.* [P1 Private Hospital Site B]

Overall, discharge was perceived as being better managed for those people who had had minor procedures while those who had more serious procedures or conditions felt there could have been more consistency-of-care and better support. Knowing what to expect from discharge was explained to be important, for example having your medications discussed and knowing how to use them once you got home. Pain control was also reported to be an important factor.

Patients wanted to be confident about what to expect and how to manage their expectations. In addressing aim 2, communication around discharge seemed an important factor in assessing negative and positive patient experience. If communication was unclear and instructions were difficult to understand, especially if: ‘*English was not their* [the patient’s] *first language’* [P2 Private Hospital Site B] patients were more likely to put a negative slant on their discharge experience. Conversely, if information was forthcoming and presented clearly, experiences were likely to be more positive. Positive comments described how nurses supported patients to manage their own needs post-surgery, and were attentive and coordinated in their care, listening to each other and to the patient:

*As far as being ready to be discharged, I’ve got no problems with that. I’ve felt quite comfortable and I knew what to do if something happened, so the two registered nurses, who were mainly on my ward, were very good… they told me everything I needed to know, yes.* [P2 Private Hospital Site B]

Other positive factors influencing their experience, included community staff who had seen them in hospital and then came to their homes afterwards to support them, or receiving assistance arranging taxis for outpatient appointments. There were mixed responses regarding whether post-hospital phone-calls were an expectation.

#### Theme 3: Communication between staff

In addressing the third theme, there was a similar response from all 30 focus group participants, who recommended that communication between staff and coordination between staff be considered as separate survey items. One participant [P10 Public Hospital Site A] had the experience of their discharge summary being sent to the wrong community-based service, demonstrating poor co-ordination, yet said the mistake was quickly rectified and the content was still accurate.

In addressing aim 2, a number of factors were described as influencing positive experiences, including staff frequently checking on patients and, enabling good handovers with effective information, thus removing the need for patients to retell their story to every new staff member. Doctors and nurses talking outside the patient’s room was seen as both a positive and a negative thing. Some thought it indicated seamless and ‘*excellent’* care, giving patients privacy when they needed it:

*So obviously the communication that was happening outside of my room was excellent and the doctor was always kept up to speed and the nurses were always quite attentive to any change... and reported that back*. [P1 Public Hospital Site A]

*I don’t want them to come in, just keep them away. Just leave me alone.* [P1 Private Hospital Site B]

*They don’t ask you to repeat yourself*. *Like I can quote other hospitals that I have been in where you really don’t feel like telling the same story.* [P2 Private Hospital Site C]

Others perceived this to be problematic, especially when a patient wanted to be part of the discussion.

Patients were often worried about a general lack of response to their concerns and informal complaints. This was particularly the case when complaints were about the environment (see Theme 7 for more detail), irrelevant, out-of-date information about patient treatments, indicating a failure to communicate effectively, and a lack of staff skill.

Some participants felt doctor-nurse communication did not always work well and that some doctors put up a barrier by being unapproachable. Participants generally agreed that good communicative practices included: good manners, nurses comfortably approaching doctors to exchange accurate information independent of individual staff personalities, efficient handovers, timely resolution of patient concerns and formal or informal complaints, and the inclusion of patients in dialogue.

#### Theme 4: Including patients in decisions about treatment

In addressing the fourth theme, 14 focus group participants discussed patient involvement in the decision-making process. Differences of opinion over what qualified as a decision abounded. For example, one participant described having had a routine elective surgery, where no decisions were necessary:

*For me, personally, that decision was made five months before I came in. So, all of the, you know, the surgeon describing what they’re going to do, why they’re going to do it, how they’re going to do it. All those things were a long time before anything happened here... But while I was here, there was no further conversation they just did what they said they were going to do.* [P3 Private Hospital Site B]

Participants who thought that there were many decisions to be made did not feel that decisions had to always involve patients:

*They can’t ask every question. Like, is it okay if I give you a Panadol? ... otherwise the whole place could be a mess.* [P1 Private Hospital Site B]

Overall, participants were comfortable with doctors and nurses making decisions on their behalf, as long as they explained the situation and their options clearly. Participants praised useful and timely communication and responsiveness to personal preference and discredited not being heard and not being acknowledged as a partner in the decision-making process. One participant argued that involvement in decision making was an illusion, especially when a patient was on a specific clinical pathway with a procedural process: *If I had been asked I would have said, no way, but I wasn’t asked.* [P4 Public Hospital Site C], while a large number of participants agreed that it only took one staff member to communicate ineffectively to have a devastating impact on patients. One patient’s carer recounted how both patient and carer were left out of the decision-making process and misled, with the patient becoming ill as a result:

*She was allergic to certain medications, so they gave them to her … Like, we weren’t involved in anything, we were just forced to live with whatever decisions they made, you know?* [P7 Public Hospital Site A]

#### Themes 5 & 6: Communicating with families and involving them in decision-making

In addressing the fifth and sixth themes, the item relating to family involvement by staff in decision making was discussed by all focus group participants, with similar issues raised across all four focus groups. Participants felt that when patients were well-informed, when there was no family member or caregiver present, or when a family was too overwhelmed to take in new information, family input was unnecessary. Participants said that they often wanted to be the ones to decide whether a family member should be involved in decision making. Some respondents pointed out that staff rarely appeared to feel obliged to talk to family members in these instances: *They’re very cautious and prudent. You know, I mean, they don’t volunteer information* [P6 Public Hospital Site B].

One participant felt more direction and instruction should be offered, especially for those family members responsible for the patient’s care at home, while another participant noted that there was little discussion around who was available at home to support the patient on discharge, with the recognition that external caregivers may be necessary.

A slightly different perspective on these issues was that, at times, patients needed to rely on the communication and advice of family members to understand what was going on and what decisions should be made:

*If it wasn’t for the knowledge of my family, I would really be struggling to – as a single father, to understand and to acknowledge the normal process… as a first time – as a first rounder – this is the first major operation I ever had in my life, in 56 years. There was a fair bit of paperwork. I wanted to – I wanted to make it as successful as possible and put myself in the best position possible. And yeah, I just – I think that – that there’s always room for improvement. It’s not just a busting numbers game.* [P3 Public Hospital Site A]

Overall, participants felt that patients had very different circumstances that need to be taken into account when communicating with families. What was paramount was the upholding of confidentiality while a judgement could be made about the appropriateness of sharing information and decision-making responsibilities. Therefore, any items relating to family involvement should be dependent on not only the intentions of involving family members in a case, but also whether this worked for all patients in all situations:

*By all means, if I was too unwell to communicate, then go for it. [P2* Public Hospital Site C*]*

#### Theme 7: Response to patient concerns and complaints

Staff response to patient concerns or complaints was discussed by 16 participants. Some noted that the wording of this item may need to change as there was a difference between staff responding to a complaint and the cause of the concern being rectified. Participants also discussed, in relation to aim 2, the impact of formal or informal complaints that were made after a patient’s stay was over. Positive experiences of well-managed complaints (made over the telephone or made more formally in writing) were limited, though having a Nurse Unit Manager around to hear a complaint and thoroughly investigate it was highly recommended:

‘*After I got home they were still phoning every day and god knows what, and I thought that was very good’.* [P5 Public Hospital Site C].

Problems of non-response to call bells (one participant suggested there should be one for emergencies and another for non-urgent requests), and the need for better behaviour from other patients were key issues raised:

*One fellow who used to - if they didn't come and answer his buzzer he'd yell, and everyone else could hear him, and he'd be going right off the deep end because no-one had come to see him.* [P9 Public Hospital Site C]

In addition, there was a lack of response to complaints about the environment, including rooms that were too warm, too cold or too noisy. Some participants noted that there had been things about their stay which had warranted a complaint but that they had not made one, recognising that: *‘nothing is 100%’* and that complaining would do nothing to alleviate the situation [P1 Public Hospital Site C].

Some participants felt some concerns could not be easily addressed when there were logistical problems involved, such as moving a disruptive or distressed patient to another room. Others saw problems with complex resolutions due to hospital protocols, such as accommodating different patients’ needs (one hard-of-hearing patients wants a television left on because he has paid for it, while another wants it turned off as it is loud and a disturbance).

In summary, many participants emphasised that acknowledging a patient’s concerns was extremely important, and that the process of managing patient concerns needs further consideration. Participants explained that follow-up phone calls allowed staff members to discuss concerns with patients and address any complaints, which was difficult to do on a ward where the person you wanted to complain about was the person looking after you, and where you could feel particularly vulnerable.

#### Theme 8: Skills and behaviours

This theme was added, in relation to participants’ responses to aim 2, as staff skills and behaviours were mentioned in all four focus groups and appeared to be significant factors that influenced patient experiences. They included behaviours such as rudeness, being dismissive, and lacking honesty, and professional groups not always: ‘*knowing what was going on’* [P3 Public Hospital Site C]*.*

Participants also discussed inconsistent care provision, such as inadequate pain relief, which came in many forms. For example, one participant described having to wait for analgesia, or having *‘adequate’* pain killers replaced prematurely by inadequate ones. Agency staff were perceived by some participants as less skilled and informed than permanent staff, while it was considered the ‘*luck of the draw’* as to who you got caring for you.

Further discussions that related to aim 2 included the positive experiences that several of the participants described in relation to attentive nurses and smooth-running services as well as attention to families’ needs. This contrasted with others’ descriptions of poor care, characterised by disrespectful, harmful or questionable practices. For some respondents, doctors could be seen as elusive, dismissive and excluding patients during ward rounds.

## Discussion

In this study, we aimed to understand patient satisfaction through clarification of user experience of hospital care across four Australian hospitals. We also aimed to clarify, through thematic analysis, factors influencing satisfaction. By considering the views of 30 patients’ responses to focus group questions, we delved more deeply into patients’ views and feelings than an initial Likert scale-based Press Ganey Patient Satisfaction Survey could achieve.

The study has drawn attention to a number of issues. Spiritual needs are less frequently at the forefront of patients’ minds than emotional needs. Environments are a source of emotional stress for patients, especially noisy spaces, lights and televisions left on, uncomfortable room temperatures, and shared rooms. There was a clear expectation among patients of high-quality information for not only patients, but also family members and carers at arrival and discharge. Good communication between staff members, and between staff and patients will result in doctors understanding more clearly what patients want but when communication breaks down, patients can feel frustrated, isolated or disempowered. A phone call, after a patient has been discharged from hospital, and follow-up support for patients and their families in their own homes, enables patients and family members to adjust to recuperation in the home and opens the way to responding to any problems post-discharge. Consistent practices, at a high standard, with shared information and staff respectful of patients’ needs, could make a significant difference to a patient’s sense of patient-centred care. Inconsistent staff communication is exacerbated by bad manners, lack of direct staff interaction and personality clashes, and patient experience is significantly mediated by staff members who do not respond to patients’ complaints.

A core message to emerge from this research is that patients can very clearly express their needs and wants following discharge from hospital. We found that if appropriate and timely information is provided to patients by caring and attentive staff members, this can help empower patients and lead to them feeling more confident about their care and about the shared decision-making process. This is echoed in the literature on patient–healthcare provider communication, with a number of studies emphasising that “whole person” knowledge, attentive clinicians and good clinician-communicators can vastly improve adherence to treatment regimes [15], belief in clinical processes, and reduce inpatient care complications [16].

When a patient recalled a particularly good or a particularly poor experience, it became the focal point around which much of what they then went on to talk about, subsequently revolved. Thus, recollection of an excellent example of care for example, or an uncaring staff member affected their whole stay and their overall impressions. This is consistent with previous work which found patient’s perceptions of care to be an important determinant of experience [8]. Furthermore, the kindness and responsiveness of the hospital’s nurses and doctors were important turning points in the minds of patients and can act as salient predictors of a good patient experience. The work of Saman et al. (2013) supports this view, and indicates that the responsiveness of staff is directly linked to a reduction in hospital-borne infection and as a result, safer environments of care [17]. This in turn can positively impact on a patient’s perception of the safety culture of a hospital. The authors note a clear link between positive associations with staff behaviour and more appropriate environments within which to recuperate from a wide range of hospital procedures. It is worth noting, however, that patients who felt less dependent on the good behaviour of staff, such as those coming to hospital to be treated for something minor, or for a shorter period of time, generally had a more positive experience of their care, regardless of the kindness and attentiveness.

Clearer information that was well-defined, and shared care plans also went a considerable way to boost patient confidence in decision-making and staff involvement in their care, while patients were keen to be part of the decisions made, regarding who else should be privy to ongoing care planning. Information that is clear, non-conflicting, and well-presented has already been identified as a core item for inclusion in patient experience questionnaires and as such, an important measurement of all patient-facilitated assessment of quality of care [18].

This paper takes the literature forward on patient satisfaction by evidencing the value and importance of user experience of hospital care, collected through qualitative, in-depth investigation. While patient satisfaction questionnaires are widely used and adapted, such as the Patient Satisfaction Questionnaire – Short Form (PSQ-18) [19], there may still be limitations to a survey [20]. For example, by their very nature, these kinds of data collection techniques provide brief and specific findings, while many measures are designed to enhance their use across a range of settings. Consequently, they often concentrate on ease of scoring and speedy completion [21], over detailed consideration and explanation of the situation under review. Furthermore, patients have been shown to present differently when asked to provide qualitative or quantitative data on satisfaction [22]. As a result, the literature supports our view that qualitative patient satisfaction data should be considered in their own right.

### Strengths and weaknesses of the study

Focus groups provided an ideal methodology for this study, matched to its purpose. Data were collected on specific understandings of the survey items from across private and public hospital patients, from three different cities. Participants were chosen from recent inpatients who are appropriate informants. Participants’ positive and negative views of treatment and care were represented across survey responses, providing balance to each group’s overall perspective. Focus group convenors used a semi-structured schedule to ensure questions were asked in the same way for each group, and that all topics were addressed. Strategic recruitment of more participants than was needed for each focus group met the challenge of high attrition rates and gave optimal numbers for three of the four groups (*n* = 7–10) and a smaller but still adequate number for the fourth (*n* = 4).

A limitation of the study was that it was based on the views of a relatively small number of patients. The diversity of experiences and the diverse meanings attached to even similar experiences meant that complete saturation of concepts was not reached. Furthermore, although many of the patients could recall and provide extensive examples of their hospital stay, due to the six-month gap, in some cases between questionnaire completion and recruitment and running of focus groups, there was no way to guarantee the veracity of their responses to each of the questions asked.

## Conclusions

This study has provided rich data on the issues leading to a positive or challenging patient experience, defining more clearly the dimensions of patient satisfaction and patient-centred care. Patient-centred care is reliant on careful planning and the considered involvement of patients from before they enter a hospital setting to their transition back into the community. While the hospital environment is important to patient’s experience, good communication is critical. Moreover, awareness of both the positive and negative effects of staff involvement on patients’ emotional, physical and mental wellbeing is paramount, with systems in place to ensure greater patient involvement in decision-making. Strategies to facilitate improved understanding of patient preferences with respect to involving family members or caregivers in decision-making are also important. Within a ‘shared decisions’ setting, patients ask to be alerted to the hospital’s complaints procedure, without fear of repercussion, including the reporting of unsafe practices and uncaring and disrespectful experiences (including those witnessed by other patients). Patients should not be fearful of jeopardising their own care or have the sense that speaking out will be ineffectual. Recommended changes will lead to greater confidence in the hospital complaints systems.

## Abbreviations

P1, P2, …. P9: Participant 1, Participant 2, Participant 3…. Etc.
PSQ – 18: Patient Satisfaction Questionnaire – Short Form
SVHA: St. Vincent Health Australia
